# Iron-Based Metal-Organic Frameworks as Multiple Cascade Synergistic Therapeutic Effect Nano-Drug Delivery Systems for Effective Tumor Elimination

**DOI:** 10.3390/ph17060812

**Published:** 2024-06-20

**Authors:** Heming Zheng, Guanghui An, Xiaohui Yang, Lei Huang, Nannan Wang, Yanqiu Zhu

**Affiliations:** 1 State Key Laboratory of Featured Metal Materials and Life-Cycle Safety for Composite Structures, MOE Key Laboratory of New Processing Technology for Nonferrous Metals and Materials, and School of Resources, Environment and Materials, Guangxi University, Nanning 530004, China; 2115301097@st.gxu.edu.cn (H.Z.); 2115301001@st.gxu.edu.cn (G.A.); y.zhu@exeter.ac.uk (Y.Z.); 2 Department of Radiation Oncology, Guangxi Medical University Cancer Hospital, Cancer Institute of Guangxi Zhuang Autonomous Region, Nanning 530021, China; 3 School of Stomatology, Minzhu Clinic of Stomatology Hospital Affiliated to Guangxi Medical University, Nanning 530007, China; 2105170252@st.gxu.edu.cn; 4 College of Engineering, Mathematics and Physical Sciences, University of Exeter, Exeter EX4 4QF, UK

**Keywords:** metal organic framework, chemodynamic therapy, photodynamic therapy, starvation therapy

## Abstract

Efforts have been made to improve the therapeutic efficiency of tumor treatments, and metal-organic frameworks (MOFs) have shown excellent potential in tumor therapy. Monotherapy for the treatment of tumors has limited effects due to the limitation of response conditions and inevitable multidrug resistance, which seriously affect the clinical therapeutic effect. In this study, we chose to construct a multiple cascade synergistic tumor drug delivery system MIL−101(Fe)−DOX−TCPP−MnO_2_@PDA−Ag (MDTM@P−Ag) using MOFs as drug carriers. Under near-infrared (NIR) laser irradiation, 5,10,15,20-tetrakis(4-carboxyphenyl)porphyrin (TCPP) and Ag NPs loaded on MDTM@P−Ag can be activated to generate cytotoxic reactive oxygen species (ROS) and achieve photothermal conversion, thus effectively inducing the apoptosis of tumor cells and achieving a combined photodynamic/photothermal therapy. Once released at the tumor site, manganese dioxide (MnO_2_) can catalyze the decomposition of hydrogen peroxide (H_2_O_2_) in the acidic microenvironment of the tumor to generate oxygen (O_2_) and alleviate the hypoxic environment of the tumor. Fe^3+^/Mn^2+^ will mediate a Fenton/Fenton-like reaction to generate cytotoxic hydroxyl radicals (·OH), while depleting the high concentration of glutathione (GSH) in the tumor, thus enhancing the chemodynamic therapeutic effect. The successful preparation of the tumor drug delivery system and its good synergistic chemodynamic/photodynamic/photothermal therapeutic effect in tumor treatment can be demonstrated by the experimental results of material characterization, performance testing and in vitro experiments.

## 1. Introduction

Despite the rapid development of the medical technology level in the past decades, malignant tumors are still one of the most difficult diseases to cure, seriously endangering human health and life [1,2,3]. Conventional tumor treatments are based on surgical resection, radiotherapy and chemotherapy. As conventional treatment often fails to achieve the desired therapeutic effect, its high cost and the side effects that cannot be ignored also make malignant tumors a heavy burden for society and the patient’s family [4,5,6,7]. With the development of nanomaterials, a series of more effective tumor treatments have emerged, including chemodynamic therapy, photodynamic therapy, photothermal therapy, etc. [8,9,10]. They have attracted widespread attention as novel treatments due to their excellent efficacy, low side effects and low cost.

Metal–organic frameworks (MOFs) are a class of organic–inorganic composites with diverse functionalities [11,12]. MOFs can achieve the conditions of high drug loading, low toxicity, ability to control drug release, targeted drug delivery, easy-to-engineer structures, and ability to be detected by imaging tools, which are required for an ideal drug delivery system [13,14,15,16]. Therefore, MOFs are well suited for drug delivery studies and have the potential to be applied in the field of anticancer drug delivery. Among these MOFs, MIL−101(Fe) has unique advantages as an iron-based MOF prepared with Fe^3+^ as the metal center, including low cost, Fenton reaction capability, porousness, and biodegradability for oncology therapy [17,18,19]. In particular, the redox activity of MIL−101(Fe) in the tumor microenvironment makes it an ideal candidate for tumor therapy. Fenton/Fenton-like reaction is the process of generating ·OH from H_2_O_2_, catalyzed by iron or other metal ions [20,21,22]. Chemodynamic therapy (CDT) is an effective treatment based on the endogenous Fenton/Fenton-like reaction, which catalyzes the generation of highly cytotoxic ·OH from H_2_O_2_, thereby promoting apoptosis [23,24,25]. The selection of MIL−101(Fe) as a drug carrier provides an abundant source of iron for chemodynamic therapy. MnO_2_ is sensitive and degradable in the tumor microenvironment and can decompose endogenous H_2_O_2_ into O_2_, alleviating the hypoxic environment of tumors and providing raw material for the conversion of highly toxic ROS for photodynamic therapy [26,27,28]. More notably, MnO_2_ is also a potent GSH-depleting agent and is capable of releasing low intrinsic toxicity Mn^2+^, which facilitates a Fenton-like reaction, thus enhancing the therapeutic efficacy of chemodynamic therapy [29,30].

Innovative non-invasive phototherapies based on NIR irradiation have received much attention, with photodynamic therapy and photothermal therapy as representative therapies. Specifically, photosensitive nano-systems, including photosensitizers and photothermal converters, can convert NIR laser irradiation into chemical or thermal energy, generating large amounts of cytotoxic ROS or a large amount of heat to cause destructive damage to targeted tissues, thus achieving the goal of killing tumor cells [31,32]. Photodynamic therapy (PDT) achieves site-specific vascular occlusion and cell destruction with minimal damage to the adjacent normal tissues by combining photosensitizers with low-power, long-duration NIR lasers [33,34,35]. The therapeutic efficacy of chemodynamic and photodynamic therapy is dependent on the tumor microenvironment, and combining the small molecule photosensitizer TCPP with MIL−101(Fe) can achieve a combined potentiation of tumor therapy. Photothermal therapy (PTT) is a non-invasive local tumor treatment modality that relies on a photothermal converter to generate sufficient heat under near-infrared light irradiation to inhibit or even ablate tumors [36,37]. The efficient thermal ablation achieved by photothermal therapy relies on the photothermal conversion effect of photothermal converters. Among them, silver nanoparticles (Ag NPs), as precious metal nanoparticles with excellent optical properties, have the advantages of high light absorption, high thermal conductivity, tunability, biocompatibility and reusability, which make them ideal photothermal-converting agents for realizing highly efficient photothermal therapy [38,39,40,41].

**Scheme 1:**
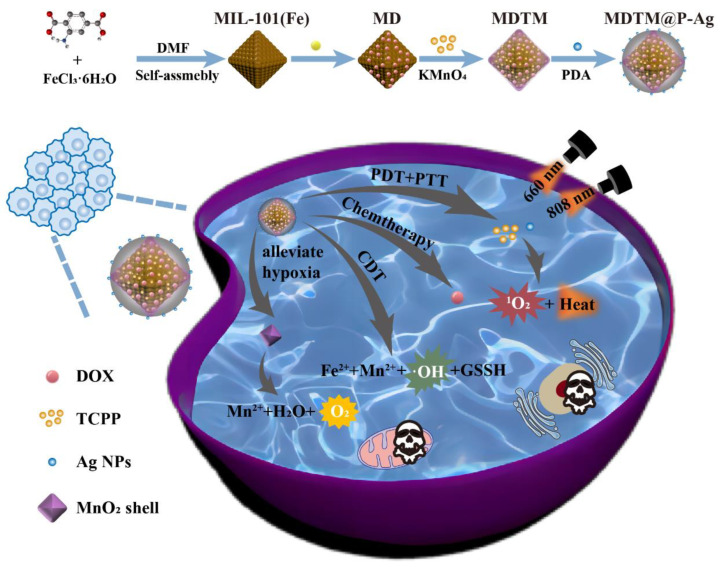
A schematic illustration of MDTM@P−Ag nano-drug delivery system and its mechanisms of multiple cascade synergistic chemodynamic/photodynamic/photothermal therapy.

In this study, MIL−101(Fe) was chosen as the drug carrier to construct a nano-loading system for multimodal combined therapy combining chemodynamic therapy, photodynamic therapy and photothermal therapy. As shown in Scheme 1, MIL−101(Fe) was used as a carrier to provide an iron source for inducing a Fenton reaction and loaded with doxorubicin hydrochloride (DOX) and the photosensitizer TCPP. Then, MnO_2_ NPs were encapsulated on the surface of the nanoparticles by the redox reaction of potassium permanganate (KMnO_4_), followed by encapsulating polydopamine (PDA) in the outermost layer and loading Ag NPs. The synthesized triple synergistic drug-carrying nanoplatform was MIL−101(Fe)−DOX−TCPP−MnO_2_@PDA−Ag (MDTM@P−Ag), which is expected to be aggregated at the tumor site through passive targeting by the enhanced permeability and the retention (EPR) effect. Upon reaching the tumor site, the PDA shell and MIL−101(Fe) were cleaved in the acidic microenvironment within the tumor, leading to drug release. Subsequently, MnO_2_ could catalyze the decomposition of H_2_O_2_ in the acidic microenvironment of the tumor to generate O_2_ and alleviate the hypoxic environment of the tumor. The generated Mn^2+^ could further undergo a Fenton-like reaction with the endogenous H_2_O_2_ and consume the GSH in the tumor, thus promoting the chemodynamic therapeutic effect. Under 660 nm and 808 nm NIR laser irradiation, TCPP and Ag NPs can be activated to realize combined photodynamic/photothermal therapy. TCPP can induce the molecular oxygen in the tumor microenvironment to be converted into cytotoxic ROS under 660 nm laser irradiation to effectively induce the apoptosis of the tumor cells, thus realizing photodynamic therapy. Ag NPs can realize photothermal conversion under 808 nm laser irradiation, thus greatly destroying tumor tissues with insufficient heat dissipation ability and realizing photothermal therapy. In summary, MDTM@P−Ag effectively realized the multimodal synergistic treatment of CDT/PDT/PTT, which exerted a stronger therapeutic effect compared to the single mode of treatment.

## 2. Result and Discussion

### 2.1. Synthesis and Characterization of MDTM@P−Ag

**Figure 1:**
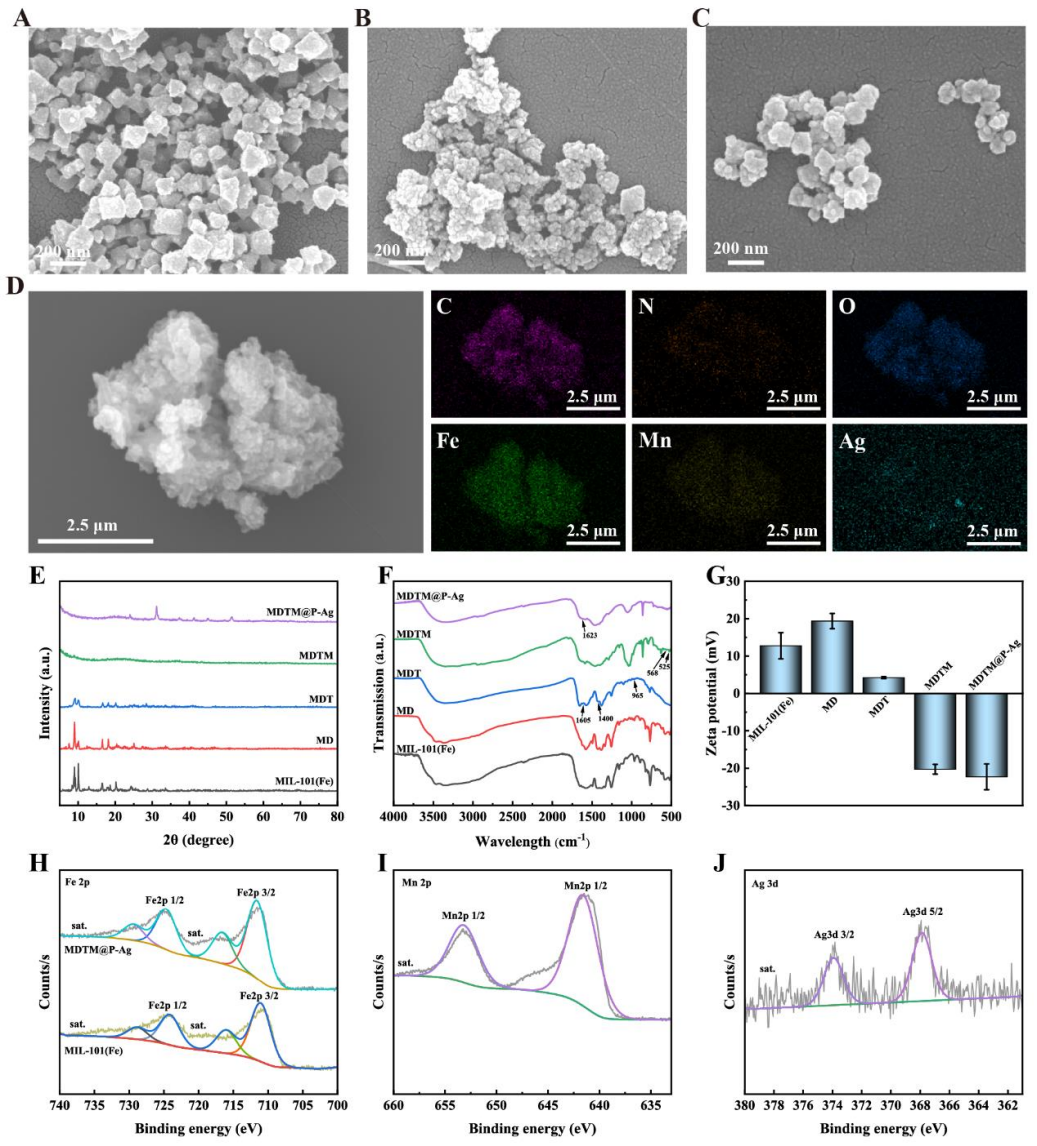
SEM images of (**A**) MIL−101(Fe), (**B**) MDTM, (**C**) MDTM@P−Ag. (**D**) SEM image of MDTM@P−Ag and elemental mappings of the corresponding SEM image. (**E**) XRD patterns of MIL−101(Fe), MD, MDT, MDTM, MDTM@P−Ag. (**F**) FT-IR spectra of MIL−101(Fe), MD, MDT, MDTM and MDTM@P−Ag. (**G**) Zeta potentials of MIL−101(Fe), MD, MDT, MDTM, MDTM@P−Ag (*n* = 3). (**H**) XPS spectra of the Fe element in MIL−101(Fe) and MDTM@P−Ag. (**I**) XPS spectra of the Mn element in MDTM@P−Ag. (**J**) XPS spectra of the Ag element in MDTM@P−Ag.

The synthesis of MDTM@P−Ag has been briefly demonstrated in Scheme 1. As shown in Figure 1A, it can be observed from the scanning electron microscope (SEM) image of MIL−101(Fe) that MIL−101(Fe) showed an angular octahedral morphology, with an average particle size of about 150 nm, which was as same as in the previous literature [42,43]. The MIL−101(Fe) NPs were well dispersed, which could be used as good drug carriers. As shown in Figure S1, after loading the drug DOX and photosensitizer TCPP, the morphology of MDT did not change significantly compared to MIL−101(Fe), indicating that the loading of DOX and TCPP did not have a significant effect on the morphology of MIL−101(Fe). After the surface coating of the MnO_2_ NPs, it can be observed that the surface of MDTM was slightly rough, and the average particle size increased to about 175 nm, which initially proved the successful coating of the MnO_2_ NPs (Figure 1B). As shown in Figure 1C, it can be observed that the morphology of MDTM@P−Ag tended to be rounded and the average particle size was around 200 nm, which was still well dispersed, proving the successful preparation of MDTM@P−Ag. Energy-dispersive X-ray spectroscopy (EDS) showed the distribution of different elements in MDTM@P−Ag (Figure 1D). The uniform distribution of elements of C, N, O, Fe, Mn and Ag in MDTM@P−Ag proved the successful synthesis of MIL−101(Fe) as well as the effective loading of the MnO_2_ NPs and Ag NPs on MIL−101(Fe), which in turn proved the successful preparation of MDTM@P−Ag.

The particle size distribution and stability of the MDTM@P−Ag nanocarrier platform were evaluated by a dynamic light scattering (DLS) test (Figure S2). Under the dispersion conditions of deionized water, PBS buffer, DMEM medium and DMEM + 10% fetal bovine serum (FBS) complete medium, the MDTM@P−Ag were well dispersed, presenting as a well-dispersed brown suspension (Figure S2A). The results, as shown in Figure S2B, indicated that the hydrated particle size of MDTM@P−Ag NPs was about 272 nm and remained stable after six days of incubation under the above culture conditions. This indicates that no significant aggregation of MDTM@P−Ag occurred, laying the foundation for further in vitro experiments. Compared with the SEM measurements, the DLS-measured size of MDTM@P−Ag was slightly larger, a result that may be attributed to the hydration swelling effect.

As shown in the powder X-ray diffraction (PXRD) patterns in Figure 1E, the XRD pattern of the synthesized MIL−101(Fe) was in perfect agreement with the literature report [43], which proved the successful synthesis of MIL−101(Fe). The characteristic peaks in the XRD patterns of both MD and MDT were similar to MIL−101(Fe), indicating that the crystal structure and crystallinity of MIL−101(Fe) were retained after loading with DOX and TCPP. As shown in Figure S3, the XRD pattern of MDTM showed a characteristic peak at 2θ = 36.7°, indicating that the capped MnO_2_ NPs may have a δ−MnO_2_ (JCPDS 18-0802) crystal structure [44,45]. Four characteristic peaks, centered at 38.10°, 44.25°, 64.45° and 77.40° in MDTM@P−Ag, correspond to the (111), (200), (220) and (311) planes of Ag (JCPDS 04-0783) [46], which suggests the successful loading of Ag NPs. The above XRD results demonstrate the successful synthesis of MDTM@P−Ag. The Fourier transform infrared spectra (FTIR) of MDTM@P−Ag showed characteristic absorption peaks for DOX, TCPP, MnO_2_ and PDA (Figure 1F). In the FTIR spectra of MDT, new additional peaks were found at 1605, 1400 and 965 cm^−1^, which were due to the C=O and N-H stretching vibrations of TCPP [43], indicating that TCPP was successfully covalently bonded to MIL−101(Fe). The characteristic peaks at 568 and 525 cm^−1^ appearing in the FTIR spectra of MDTM were due to the Mn−O vibrations [47]. In the FTIR spectra of MDTM@P−Ag, the characteristic peak appearing at 1623 cm^−1^ was the result of the C=C stretching vibration associated with the backbone vibration of the aromatic ring [48], confirming the presence of PDA and DOX. The FTIR results successfully confirmed that MIL−101(Fe) was loaded with DOX, TCPP and MnO_2_ NPs and encapsulated with PDA on the outer shell, the successful synthesis of MDTM@P−Ag. To analyze the elemental composition and chemical valence states contained in MDTM@P−Ag, X−ray photoelectron spectroscopy (XPS) analysis was performed. As shown in Figure S4, the XPS full spectrum of MDTM@P−Ag indicates that MDTM@P−Ag contains the elements C, N, O, Fe, Mn and Ag, which proves the existence of the MIL−101(Fe) skeleton and the successful loading of MnO_2_ NPs and Ag NPs. As shown in Figure 1H, the XPS spectra of Fe in MIL−101(Fe) and MDTM@P−Ag show satellite peaks of Fe 2p1/2 and Fe 2p2/3 at 723.61 eV and 710.37 eV, respectively, which proves the presence of Fe in MIL−101(Fe) and MDTM@P−Ag in the form of trivalent iron. As shown in Figure 1I, the satellite peaks appearing at 641 eV and 653 eV in the XPS spectrum of Mn for MDTM@P−Ag correspond to Mn 2p3/2 and Mn 2p1/2, respectively, which were characteristic peaks of MnO_2_, demonstrating that the Mn existed in the form of MnO_2_ in MDTM@P−Ag. As shown in Figure 1J, the XPS spectrum of Ag in MDTM@P−Ag shows the existence of two Ag 3d peaks with the binding energy of 368 eV and 374 eV, corresponding to the satellite peaks of Ag 3d5/2 and Ag 3d3/2, respectively, which proves that the Ag NPs were successfully loaded on MDTM@P and that MDTM@P−Ag was successfully synthesized.

The zeta potentials were tested for each step of the synthesized materials (Figure 1G). The zeta potential of MIL−101(Fe) was 12.68 mV, and after loading with DOX, the zeta potential of MD became higher at 19.36 mV. Due to the fact that the surface of the TCPP−loaded charge carried by the surface of the material is usually negative, the zeta potential of MDT decreased from 19.36 mV to 4.25 mV. After capping the MDTM with MnO_2_ NPs, the zeta potential decreased from 4.25 mV to −20.27 mV. Because of the low equipotential point of the PDA [49], the zeta potential of MDTM@P−Ag further decreased to −22.3 mV. Generally, the higher the absolute value of the zeta potential is, the greater the electrostatic repulsion between the particles, resulting in better dispersion and stability in solution. Therefore, the synthesized MDTM@P−Ag can be stable and effectively circulated in vivo and in tumor tissues. From the N_2_ adsorption–desorption isotherms in Figure S5, MIL−101(Fe) had a high specific surface area and possessed a high number of pore structures, which made it a good drug carrier. The comparison of the N_2_ adsorption–desorption isotherms of MIL−101(Fe) and MD revealed that there was a certain decrease in the specific surface area and pore space of MD, as compared to MIL−101(Fe), which suggested DOX was successfully loaded.

### 2.2. Drug Release and Catalytic Ability of MDTM@P−Ag

**Figure 2:**
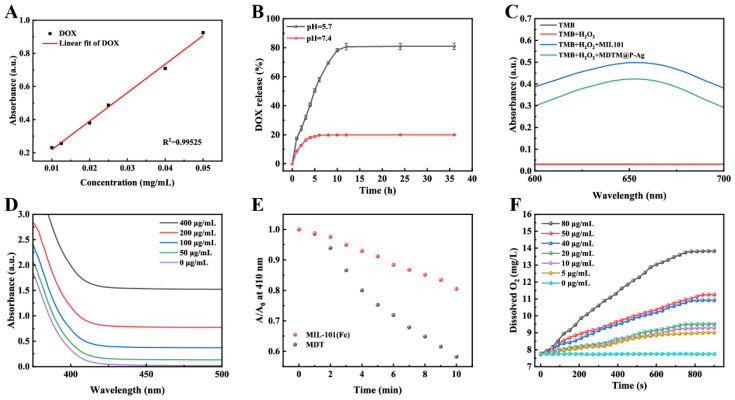
(**A**) The standard curve of DOX calculated by the absorbance at 490 nm. (**B**) The ability of MDTM@P−Ag to release DOX at different pH (*n* = 3). (**C**) The UV–vis absorption spectra of TMB without H_2_O_2_, with H_2_O_2_ and with both H_2_O_2_ and MIL−101(Fe) or MDTM@P−Ag. (**D**) The UV–vis absorption spectra of DTNB at different concentrations of MDTM treated with GSH. (**E**) The UV–vis absorption changes of DPBF at 410 nm during 10 min of irradiation with the 660 nm laser. (**F**) Capacity of different concentrations of MDTM@P−Ag to react with H_2_O_2_ to produce O_2_.

To estimate the drug loading and release capability of MDTM@P−Ag, the standard curve of DOX was detected and plotted using UV–vis absorption spectroscopy (Figure 2A). The drug loading efficiency is 39.64%, and the encapsulation rate is 5.89% for DOX in MDTM@P−Ag. The drug loading efficiency of TCPP is 80.68%. The drug release kinetics of DOX in MDTM@P−Ag was investigated, as shown in Figure 2B. The cumulative release of DOX was tested by placing the prepared MDTM@P−Ag NPs in a weak acidic condition (pH = 5.7) that simulated the tumor microenvironment. The drug release of DOX showed an increasing trend in the starting period, and when the test was carried out for about 12 h, the cumulative release of DOX reached the maximum of about 80.93%. Under neutral physiological conditions simulating normal tissue at pH = 7.4, the cumulative release of DOX reached a maximum of about 18.83% at around 5 h and remained at that lower level. When MDTM@P−Ag was placed in a lower pH environment, the drug release was faster. This indicated that the encapsulated PDA shell can be selectively cleaved under acidic conditions, suggesting that the drug release from MDTM@P−Ag was pH-sensitive, preventing the premature release of DOX in normal tissues, thus conferring targeting and controllability to the MDTM@P−Ag NPs.

The chemodynamic properties of MDTM@P−Ag were tested by TMB/H_2_O_2_ chromogenic reaction and glutathione (GSH) depletion. To illustrate whether H_2_O_2_ and the synthesized materials had any effect on the color development of 3,3′,5,5′-tetramethyl-benzidine (TMB), the groups of TMB, TMB + H_2_O_2_, TMB + H_2_O_2_ + MIL−101(Fe) and TMB + H_2_O_2_ + MDTM@P−Ag were set up. As shown in Figure 2C, no absorption peaks were detected at 652 nm in both the TMB and TMB+ H_2_O_2_ groups, whereas significant absorption peaks were detected at 652 nm in the TMB + H_2_O_2_ + MIL−101(Fe) and TMB + H_2_O_2_ + MDTM@P−Ag groups, indicating that the Fe^3+^ in the materials was released and formed ·OH in a Fenton reaction with H_2_O_2_ in the solution. It can be observed that the absorption peak of the TMB + H_2_O_2_ + MDTM@P−Ag group was low compared to that of the TMB + H_2_O_2_ + MIL−101(Fe) group, which was attributed to the fact that the MDTM@P−Ag is wrapped with the PDA shell, and the Fe^3+^ in MIL−101(Fe) wrapped by PDA was not easy to be released into the solution under neutral conditions. The intensity of the absorption peaks at 652 nm was detected for MIL−101(Fe), MDTM and MDTM@P−Ag, considering the H_2_O_2_ concentration gradient and the reaction time gradient of TMB in a weakly acidic environment simulating the tumor microenvironment at pH = 6.5. As shown in Figure S6, it can be observed that at a certain material concentration, the absorption peaks of TMB increased with the increase in the concentration of H_2_O_2_ and the reaction time, indicating that more ·OH was produced in the system. At both the same material concentration and the same concentration of H_2_O_2_, it can be observed that the absorption peaks of TMB in the MDTM group were higher than those of the TMB in the MIL−101(Fe) group. This was because MnO_2_ in MDTM could react with H_2_O_2_ and H^+^, and the generated Mn^2+^ could further react with H_2_O_2_ in a Fenton-like reaction, which promoted the generation of ·OH. As a result, the generation of ·OH in the MDTM group was greatly increased compared to the generation of ·OH by the Fenton-like reaction involving only Fe^3+^ in the MIL−101(Fe) group. In summary, the chemical kinetic performance of MDTM@P−Ag was closely related to the H_2_O_2_ concentration, the reaction time and the concentration of ions involved in Fenton/Fenton-like reaction, and the amount of ·OH produced was positively correlated with the H_2_O_2_ concentration and the reaction time.

The glutathione (GSH) depletion ability of MDTM@P−Ag can be demonstrated by the UV–Vis spectra of 5,5′-dithiobis(2-nitrobenzoic acid) (DTNB), a potent functional group for the detection of the sulfhydryl groups of GSH (-SH), which bind to GSH and show an absorption peak near 412 nm [50,51]. As shown in Figure 2D and Figure S7, the absorption peak at 412 nm appeared to decrease with the addition of MIL−101(Fe) and MDTM, indicating that both MIL−101(Fe) and MDTM can consume GSH and exhibit a concentration dependence related to the concentration of the material added. In the MDTM + GSH + DTNB group, the reaction can be promoted because the MnO_2_ in the outer layer responds well to GSH, and it can be observed that the intensity change of the absorption peak at its 412 nm was larger than that of the MIL−101(Fe) + GSH + DTNB group. The above results indicate that MDTM@P−Ag can act as a GSH-depleting agent and effectively consume GSH, which is expected to regulate the redox balance in tumor cells.

1,3-Diphenylisobenzofuran (DPBF) is oxidatively degraded in the presence of single oxygen (^1^O_2_) [43,52]. Therefore, DPBF was chosen to detect whether MIL−101(Fe) and MDT could produce ^1^O_2_ under NIR laser irradiation at 660 nm. Figure 2E shows the trend of DPBF at 410 nm for MIL−101(Fe) and MDT. The ^1^O_2_ produced by MDT under 660 nm NIR laser irradiation was time-dependent. With the prolongation of the 660 nm NIR laser irradiation time, the decrease in the absorption peak of DPBF gradually increased. Compared with the MIL−101(Fe) group, the absorption peak of DPBF in the MDT group decreased more, which indicates that the ^1^O_2_ generation of MDT was higher, suggesting that the photo-activated generation of ^1^O_2_ by MDT was mainly attributed to the successful loading of TCPP.

MnO_2_ is considered an ideal nanomaterial for oxygen generation. With the low pH and high H_2_O_2_ concentration of the tumor microenvironment, the introduced MnO_2_ NPs could catalyze the generation of O_2_ from H_2_O_2_ in the tumor microenvironment. The generated O_2_ can increase the local oxygen concentration of the tumor and alleviate the inherent hypoxic environment of the tumor [53,54,55]. To evaluate the ability of MDTM@P−Ag to catalyze the generation of O_2_ from H_2_O_2_, a series of concentration gradients of aqueous MDTM@P−Ag solution were set up. As shown in Figure 2F, when 5 μg/mL of MDTM@P−Ag was added to the reaction system containing H_2_O_2_, a small amount of O_2_ production could be observed, and the concentration of dissolved oxygen in the system amounted to 9 mg/mL after 15 min of the reaction. As the concentration of the added MDTM@P−Ag increased, the concentration of dissolved oxygen produced in the system increased in a concentration-dependent manner. When MDTM@P−Ag at a concentration of 80 μg/mL was added, a large amount of bubbles were generated, and the concentration of dissolved oxygen within the system increased rapidly, reaching a maximum value of 13.75 mg/mL at about 12 min into the reaction. The above results indicate that MDTM@P−Ag has a good ability to generate O_2_, and it has the potential to catalyze and alleviate the hypoxic environment of tumors.

### 2.3. The Photothermal Performance of MDTM@P−Ag

**Figure 3:**
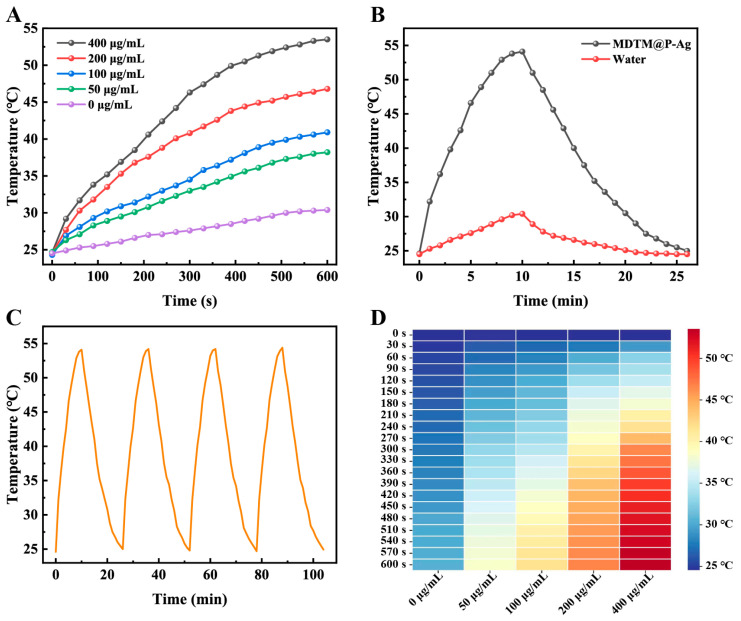
(**A**) Temperature changes of MDTM@P−Ag with different concentrations under an 808 nm laser. (**B**) Heating/cooling curves of deionized water and MDTM@P−Ag under an 808 nm laser. (**C**) Heating/cooling cycle curves of MDTM@P−Ag under an 808 nm laser. (**D**) Heat map for temperature changes of MDTM@P−Ag with different concentrations under an 808 nm laser.

NIR laser irradiation at 808 nm was chosen to study the photothermal conversion performance of MDTM@P−Ag. As shown in Figure 3A, different concentrations of MDTM@P−Ag were dispersed in deionized water and irradiated under an 808 nm NIR laser for 10 min. With the growth of laser irradiation time and the increase in MDTM@P−Ag concentration, the temperature of the system increased significantly, showing obvious time and concentration dependence. When the concentration of MDTM@P−Ag was 400 μg/mL, the temperature of the system increased from 24.6 °C to 53.5 °C in 10 min, with the largest temperature increase. As shown in Figure 3B, compared to the aqueous solution containing MDTM@P−Ag, the temperature of pure deionized water increased somewhat, but not significantly, indicating that the temperature increase of the system was mainly attributed to the photothermal conversion ability of MDTM@P−Ag. As shown in Figure 3C, the photothermal stability of MDTM@P−Ag in the aqueous solution was evaluated by four heating/cooling cycle tests. There was no significant change in the temperature after four heating/cooling cycle tests, indicating that the photothermal performance of MDTM@P−Ag remained unchanged and was photothermally stable. The temperature changes of different concentrations of MDTM@P−Ag at different times of 808 nm NIR laser irradiation can be visualized in Figure 3D. Using the data in Figure 3B and Equations (3) and (4), the photothermal conversion efficiency η of MDTM@P−Ag is 61.41%. The above experimental results demonstrate that MDTM@P−Ag has excellent and stable photothermal properties and could be used as a photothermal agent for the photothermal treatment of tumors.

### 2.4. In Vitro Therapeutic Effects of MDTM@P−Ag

**Figure 4:**
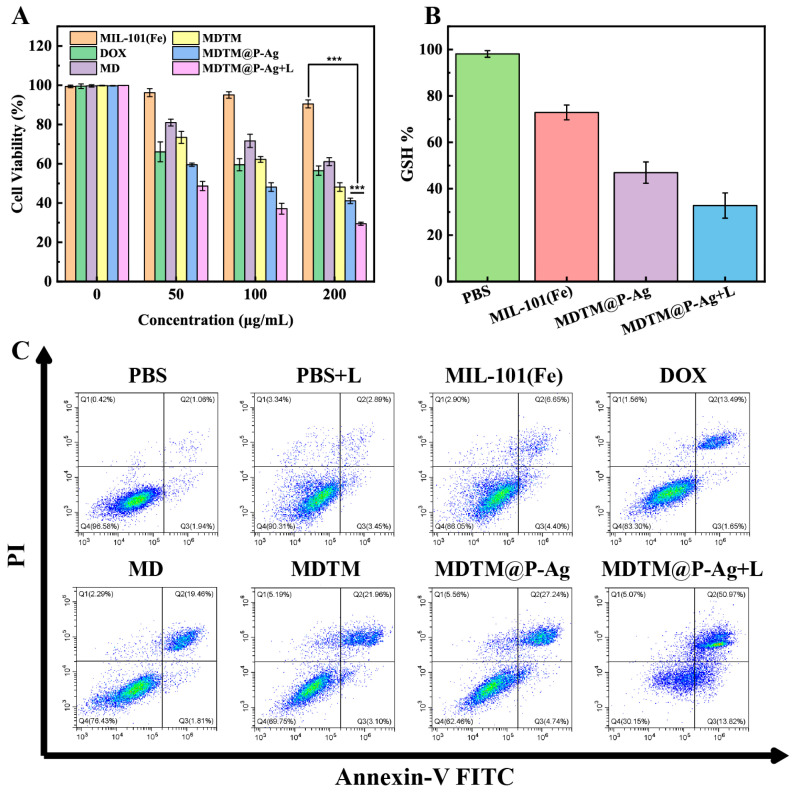
(**A**) The cell viability of HepG2 cells after treatment with different concentrations of different materials. (**B**) GSH contents in HepG2 cells after treatment with PBS, MIL−101(Fe), MDTM@P−Ag and MDTM@P−Ag + L, respectively. (**C**) Flow cytometry analysis of HepG2 cells after treatment with different materials, determined by Annexin V-FITC/PI staining (*** *p* < 0.001. *n* = 3).

To evaluate the synergistic therapeutic effect of MDTM@P−Ag-induced CDT/PDT/PTT on HepG2 cells, the cell viability of HepG2 cells after treatment with different concentrations of the materials was quantitatively analyzed using the CCK-8 assay. As shown in Figure 4A, there was almost no decrease in cell viability in the MIL−101(Fe) group, indicating that MIL−101(Fe) was non-cytotoxic, did not cause significant damage to HepG2 cells and could be safely used as a drug carrier. The cytotoxicity of HepG2 cells was significantly decreased in the MD and MDTM-treated HepG2 cells, with more decrease in the MDTM group, which was attributable to the Fe^3+^/Mn^2+^-mediated chemodynamic therapeutic effect, which was superior to the Fe^3+^-mediated chemodynamic therapeutic effect alone. The killing of HepG2 cells by MDTM@P−Ag was greatly enhanced under NIR laser irradiation at 660 nm and 808 nm. When the MDTM@P−Ag concentration was 200 μg/mL, the survival rate of HepG2 cells treated with MDTM@P−Ag and NIR laser irradiation decreased to 29.44%. On the one hand, Fe^3+^/Mn^2+^ could mediate efficient chemodynamic therapy, and MnO_2_ could also react with endogenous H_2_O_2_ to generate O_2_, which could effectively alleviate the hypoxic environment in the tumor cells. On the other hand, TCPP and Ag NPs could be activated by the NIR laser at 660 nm and 808 nm, respectively, to generate cytotoxic ROS while realizing photothermal conversion to induce apoptosis and necrosis in HepG2 cells. This suggests that MDTM@P−Ag can be well combined with chemodynamic therapy, photodynamic therapy and photothermal therapy, with a good synergistic therapeutic effect in HepG2 cells.

The depletion of GSH by the nanocarrier platform MDTM@P−Ag was investigated. As shown in Figure 4B, the intracellular GSH levels in HepG2 cells treated with different materials were decreased in every group of HepG2 cells after incubation with the drug. Compared with the PBS group, the intracellular GSH level in the MIL−101(Fe)-treated group decreased to 72.9%, which was attributed to the reaction of Fe^3+^ in MIL−101(Fe) with the intracellular GSH, leading to the depletion of GSH, indicating the excellent chemodynamic properties of MIL−101(Fe). MnO_2_ in MDTM@P−Ag can also react with the GSH in cells to form glutathione disulfide and Mn^2+^, leading to a large depletion of GSH. As a result, the intracellular GSH level decreased more in the MDTM@P−Ag-related treatment group, whereas the intracellular GSH level decreased to 32.75% in the MDTM@P−Ag+L group. The above results further demonstrate the excellent chemodynamic therapeutic properties of the synthesized MDTM@P−Ag.

The effect of MDTM@P−Ag on the apoptosis of HepG2 cells was investigated by flow cytometry with Annexin V-FITC/PI double staining. As shown in Figure 4C, Q1, Q2, Q3 and Q4 represent dead cells, late apoptotic cells, early apoptotic cells and live cells, respectively. Except for the PBS group, all groups showed different degrees of apoptosis, with cells migrating from Q4 (live cells) to Q2 (late apoptotic) and Q3 (early apoptotic). The ratio of dead and apoptotic cells in the PBS group, the PBS + L group and the MIL−101(Fe) group was maintained at a low level, demonstrating that MIL−101(Fe) had good biocompatibility. There was no negative effect on the normal growth of the HepG2 cells, and the NIR laser irradiation had no effect on the normal growth of the HepG2 cells. The late apoptosis rate of the cells in both the DOX and MD groups showed a certain increase. Compared with these two groups, the MDTM group increased the production of O_2_ in the tumor cells, resulting in a late apoptosis rate of 21.96%. Due to the successful loading of the MnO_2_ NPs, MDTM could effectively alleviate the hypoxic environment in the tumor cells and at the same time enhance the Fenton/Fenton-like reaction and GSH consumption in the tumor cells, which greatly improved the synergistic therapeutic effect, leading to a greater degree of apoptosis rate. Compared with the other groups, the apoptosis rate and mortality rate of the MDTM@P−Ag-related treatment group were the highest, with the MDTM@P−Ag + L group having the highest late apoptosis rate of 50.97%. All these results indicate that MDTM@P−Ag has excellent synergistic therapeutic effects in chemodynamic therapy, photodynamic therapy and photothermal therapy, inducing apoptosis and significantly enhancing the therapeutic effects by controlling the generation of ROS, the consumption of GSH, the photothermal conversion and the alleviation of the intracellular hypoxic environment, thus laying a good foundation for in vivo treatment. This result is consistent with that of the CCK-8 assay.

**Figure 5:**
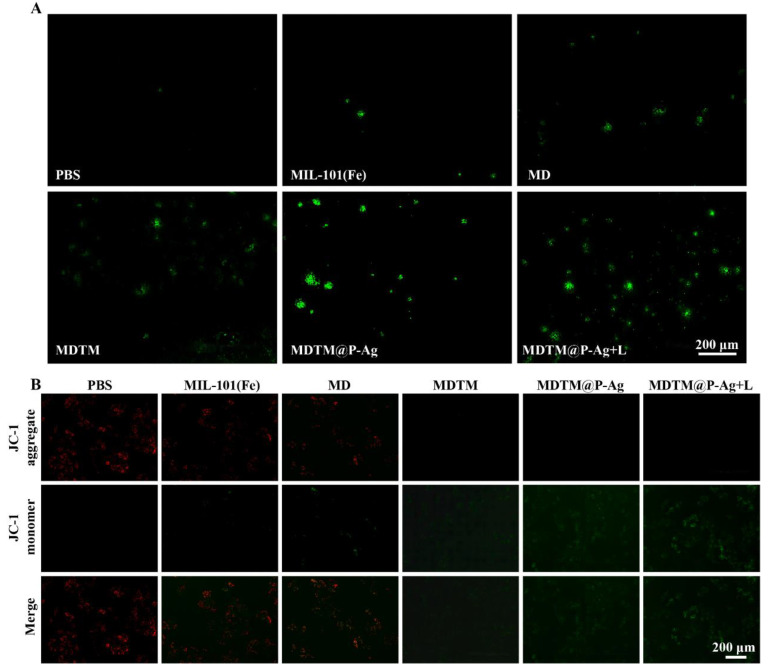
(**A**) Fluorescence images of the generation of ROS in HepG2 cells stained with DCFH-DA after treatment with different materials. (**B**) Fluorescence images of HepG2 cells stained with JC-1 after treatment with different materials (the scale bar is 200 μm).

The generation of ROS in HpeG2 cells after treatment with different materials was detected using 2′,7′-dichlorofluorescein diacetate (DCFH-DA), a fluorescent probe that emits green fluorescence when the ROS are oxidized [56]. As shown in the cell fluorescence images in Figure 5A, the HpeG2 cells in the MIL−101(Fe) group and the MD group showed weak green fluorescence. This was due to a certain cleavage of the MIL−101(Fe) skeleton in the weakly acidic environment within the tumor cells, in which there was a certain release of Fe^3+^, which reacted with the endogenous H_2_O_2_ in the Fenton reaction to generate ROS. After loading the MnO_2_ shell, MnO_2_ could react with intracellular H_2_O_2_ and H^+^ in a Fenton-like reaction, thus further promoting the generation of O_2_ and ROS, and realizing the depletion of endogenous GSH and the alleviation of hypoxia. Therefore, the more ROS were generated, the more DCFH-DA was oxidized, and the intensity of the green fluorescence was relatively higher. The HpeG2 cells in the MDTM@P−Ag-treated group showed the highest intensity of green fluorescence after the addition of NIR laser irradiation. This indicates that the MDTM@P−Ag + L group had a stronger ability of light-induced generation of ROS compared with the other groups and the MDTM@P−Ag-treated group without the addition of 660 nm near-infrared laser irradiation, which can realize good synergistic chemodynamic, photodynamic and photothermal therapeutic effects.

The mitochondrial membrane potential level of HepG2 cells after treatment with different materials was detected by JC-1 staining. The level of mitochondrial membrane potential is an important indicator of mitochondrial function. JC-1 is a specific probe for mitochondrial membrane potential, and its fluorescence is related to the mitochondrial membrane potential Green fluorescence usually represents the loss of mitochondrial membrane potential, while red fluorescence represents the normal mitochondrial membrane potential [57,58]. As shown in Figure 5B, both the PBS group and the MIL−101(Fe) group showed bright red fluorescence, indicating that the HepG2 cells were in a normal state and survived well, which proved that MIL−101(Fe) was a good drug carrier, with good biocompatibility. In the MD group, the intensity of the red fluorescence was weakened to a certain extent, and the intensity of the green fluorescence was enhanced, which indicates that there was partial apoptosis and a loss of mitochondrial membrane potential, proving that MD had a significant chemotherapeutic effect. The MDTM group showed stronger green fluorescence and a significant decrease in mitochondrial membrane potential. This effect was attributed to the successful loading of MnO_2_ NPs, which realized the enhancement of the Fenton/Fenton-like reaction, the depletion of endogenous GSH and the alleviation of hypoxia. These changes synergized the chemodynamic therapy, thereby achieving better therapeutic outcomes. The HepG2 cells treated with MDTM@P−Ag showed the highest intensity of green fluorescence compared to the other groups after the addition of NIR laser irradiation, indicating that after the addition of 660 nm and 808 nm laser irradiation, TCPP demonstrated its photodynamic therapeutic ability to generate cytotoxic ROS, and the Ag NPs achieved photothermal switching, which ultimately realized the combined treatment of chemodynamic therapy, photodynamic therapy and photothermal therapy. The combination of chemodynamic therapy, photodynamic therapy and photothermal therapy induced mitochondrial damage in HepG2 cells to the greatest extent. The above results prove that MDTM@P−Ag can synergize well with chemodynamic therapy, photodynamic therapy and photothermal therapy, providing a basis for the in vivo treatment of tumors.

## 3. Conclusions

We successfully constructed a multiple-cascade synergistic therapy for the drug delivery system MIL−101(Fe)−DOX−TCPP−MnO_2_@PDA−Ag, which combined chemodynamic therapy, photodynamic therapy and photothermal therapy. MIL−101(Fe) was used as a carrier to provide an iron source for inducing the Fenton reaction and was loaded with the chemotherapeutic drug DOX and the photosensitizer TCPP. MnO_2_ NPs were encapsulated on the surface of MIL−101(Fe) through the reaction of KMnO_4_ redox. Ultimately, PDA was encapsulated on the outermost layer and loaded with Ag NPs. In the weakly acidic tumor microenvironment, the PDA shell and MIL−101(Fe) underwent cleavage leading to drug release. Subsequently, MnO_2_ could catalyze the decomposition of H_2_O_2_ in the acidic microenvironment of the tumor to produce O_2_ to alleviate the hypoxic environment of the tumor. The generated Mn^2+^ could further undergo a Fenton-like reaction with the endogenous H_2_O_2_ and consume the GSH in the tumor, promoting the efficacy of chemodynamic therapy. Under the irradiation of a 660 nm NIR laser, TCPP could induce molecular oxygen in the tumor microenvironment to be converted into cytotoxic ROS to effectively induce the apoptosis of the tumor cells, thus realizing photodynamic therapy. Activated by an 808 nm NIR laser, Ag NPs could realize photothermal conversion, thus greatly destroying tumor tissues and realizing photothermal therapy. Together with TCPP, Ag NPs could facilitate the combination of photodynamic/photothermal therapy. Through a series of performance tests and cellular experiments, it was demonstrated that MDTM@P−Ag could effectively realize the multiple cascade synergistic treatment of chemodynamic/photodynamic/photothermal therapy. Compared with the single treatment mode, this multimodal treatment exerts a stronger therapeutic effect and provides a new idea for the preparation of a multifunctional nano-drug delivery system.

## 4. Experimental Sections

### 4.1. Synthesis of MIL−101(Fe)

First, 0.3747 g of FeCl_3_·6H_2_O and 1.1232 g of 2-aminoterephthalic acid (BDC-NH_2_) were dissolved in 30 mL of DMF, and the mixed solution was heated to 120 °C, which continued for 20 h. After the heating was completed and the solution was cooled down to room temperature, the precipitate was obtained by centrifugation with DMF and ethanol for several times. Then, the precipitate was purified and activated in ethanol at 60 °C by vacuum drying.

### 4.2. Synthesis of MIL−101(Fe)−DOX

100 mg of MIL−101(Fe) and 12.5 mg of DOX were dispersed in 40 mL of deionized water and magnetically stirred for 24 h at room temperature in the dark. At the end of stirring, the product was washed by centrifugation with deionized water three times, during which the centrifugation supernatant was collected and used to determine the drug loading and encapsulation rate of DOX. The collected product was MIL−101(Fe)−DOX (abbreviated as MD).

### 4.3. Synthesis of MIL−101(Fe)−DOX−TCPP

An amount of 15 mg of 1-Ethyl-(3-dimethylaminopropyl)carbodiimide hydrochloride (EDC), 10 mg of N-hydroxysuccinimide (NHS) and 25 mg of TCPP were added into 10 mL of DMF and stirred magnetically for 1 h away from light to mix them thoroughly, then the above mixture was added to 10 mL of DMF containing 100 mg of MD and stirred for 12 h at room temperature in the dark. The precipitate was collected after centrifugal washing with ethanol three times to obtain MIL−101(Fe)−DOX@TCPP (abbreviated as MDT).

### 4.4. Synthesis of MIL−101(Fe)−DOX−TCPP−MnO_2_

An amount of 50 mg of synthesized MDT dispersed in 40 mL of deionized water was placed on a magnetic stirrer with uniform stirring while adding 1 mL of 0.2 M KMnO_4_ solution drop by drop, stirring in the dark for 4 h, followed by centrifugal washing with deionized water three times to collect the product MIL−101(Fe)−DOX−TCPP−MnO_2_ (abbreviated as MDTM) [47,59].

### 4.5. Synthesis of Ag NPs

An amount of 45 mg of PVP was fully dispersed in 30 mL of deionized water as solution A, which was placed in an ultrasonic atmosphere at 300 W. An amount of 5.1 mg of AgNO_3_ was dissolved in 5 mL of deionized water as solution B, and 3 mg of NaBH_4_ was dissolved in 10 mL of deionized water as the solution C. Subsequently, the two solutions B and C were added simultaneously to solution A, which was in the ultrasonic atmosphere, and the sonication was continued for 30 min. After sonication, the product Ag NPs was centrifuged and washed several times with deionized water. The resulting product, a Ag NPs solution, was homogeneously dispersed in 50 mL of deionized water and stored.

### 4.6. Synthesis of MIL−101(Fe)−DOX−TCPP−MnO_2_@PDA−Ag

A total of 50 mg of MDTM was dispersed in 25 mL of Tris-HCl solution at pH 8.5, followed by the addition of 15 mg of dopamine hydrochloride, and stirred for 24 h under light-avoidance conditions. At the end of stirring, MIL−101(Fe)−DOX−TCPP−MnO_2_@PDA (abbreviated as MDTM@P) could be obtained by centrifugal washing with deionized water. A total of 50 mg of MDTM@P was dispersed in 25 mL deionized water, and 5 mL of the prepared Ag NPs solution was added and stirred for 24 h. After centrifugal washing, the product MIL−101(Fe)−DOX−TCPP−MnO_2_@PDA−Ag (abbreviated as MDTM@P−Ag) was yielded.

### 4.7. Drug Loading and Drug Release Assays

The drug loading and drug release of DOX in MDTM@P−Ag were quantified by UV–vis spectroscopy from the standard curves of DOX at 490 nm. We chose the dialysis bag with MWCO of 3.5 KDa to analyze the release of DOX. An amount of 10 mg of MDTM@P−Ag NPs was packed into the dialysis bag and immersed in the beaker with PBS buffer (pH = 5.7 and 7.4) while shaking at 100 rpm. A total of 2 mL of medium was collected at different time intervals, while the same volume of fresh PBS buffer was added into the system. All experiments were carried out three times. The drug loading efficiency and drug encapsulation rate were calculated based on the following equations:

(1) $$\mathrm{Drug}\;\mathrm{loading}\;\mathrm{efficiency}\;(\%)=\frac{M_{\mathrm{initial}\;\mathrm{drug}\;}-{\;M}_{\mathrm{drug}\;\mathrm{in}\;\mathrm{supernatant}}}{M_{\mathrm{initial}\;\mathrm{drug}}}\;\times \;100\%$$

(2) $$\mathrm{Drug}\;\mathrm{encapsulation}\;\mathrm{rate}\;(\%)=\frac{M_{\mathrm{initial}\;\mathrm{drug}}\;-M_{\mathrm{drug}\;\mathrm{in}\;\mathrm{supernatant}}}{M_{\mathrm{nanoparticles}}}\;\times \;100\%$$

### 4.8. The CDT Capacity Assays

TMB was used as an indicator assay to verify the ability of MDTM@P−Ag to produce ·OH, and its UV–vis absorption peak was at 652 nm. First, the following four groups of solutions were tested: (1) TMB; (2) TMB + H_2_O_2_; (3) TMB + H_2_O_2_ + MIL−101(Fe) and (4) TMB + H_2_O_2_ + MDTM@P−Ag. The changes in the UV–vis absorption peaks of the four groups were detected using a UV–vis spectrophotometer. Next, the concentration gradient of H_2_O_2_ and the time gradient of the reaction were set. A total of 100 μL of 1 mg/mL MIL−101(Fe) and MDTM@P−Ag solutions were added to 2 mL of PBS buffer (pH = 6.5) containing 3% H_2_O_2_ at different concentrations (10, 50, 100, 200, 400, 800 μg/mL), followed by the addition of 200 μL of TMB. Then, their UV–vis absorption peaks were detected after the reaction. In addition, the concentration of H_2_O_2_ in the system was controlled to be 800 μg/mL, while the other conditions were the same as those above, and the intensities of the UV–vis absorption peaks of the reaction of MIL−101(Fe) and MDTM@P−Ag with TMB were detected at intervals of 2, 4, 6 and 8 min.

The catalytic effect of MIL−101(Fe) and MDTM on GSH was detected by the colorimetric reaction of DTNB with GSH using 5,5′-dithiobis(2-nitrobenzoic acid) (DTNB) as an indicator. A 1 mM solution of GSH was incubated with different concentrations (0, 50, 100, 200, 400 μg/mL) of the MIL−101(Fe) solution and the MDTM solution for 1 h under light-avoiding conditions, followed by the addition of a 0.5 mM solution of DTNB. Ultimately, the UV absorption peaks were detected at 412 nm.

### 4.9. The PDT Capacity Assays

The generated ^1^O_2_ was detected using 1,3-Diphenylisobenzofuran (DPBF) as a fluorescent probe. A total of 500 μg/mL DPBF was added to an ethanol solution containing MDT (100 μg/mL). DPBF was accounted for 15% of the system volume. The solution was laser-irradiated for 10 min using an NIR laser with a wavelength of 660 nm and a power of 500 mW, and the UV absorption peak of DPBF at a wavelength of 410 nm was measured at one-minute intervals.

### 4.10. The PTT Capacity Assays

To evaluate the photothermal performance of MDTM@P−Ag, MDTM@P−Ag NPs were dispersed in deionized water to obtain aqueous MDTM@P−Ag solutions with different concentrations, and deionized water was used as the control group. An NIR laser with a wavelength of 808 nm and a power of 2 W/cm^2^ was selected, and the temperature of the system was detected by using a thermocouple thermometer. The systems containing a 400 μg/mL MDTM@P−Ag aqueous solution and deionized water were irradiated with an 808 nm laser for 10 min and cooled down to room temperature to obtain the heating/cooling curve. The temperature change of the system was recorded every one minute during the period. Then, the two systems above were also irradiated with an 808 nm laser for 10 min, four times, to obtain the heating/cooling cycle curve. The changes in the system temperature were recorded at every minute during the period.

The calculation of the photothermal conversion efficiency η is based on the following equation [60]:

(3) $$\eta =\frac{hs\;(T_{\max}-T_{\mathrm{surr}})\;-\;Q_{\mathrm{Dis}}}{I\;(1\;-{\;10}^{{-A}_{\lambda }})}$$

where h is the heat transfer coefficient, s is the surface area of the sample, T_max_ is the maximum steady state temperature, T_surr_ is the ambient temperature, Q_Dis_ represents the heat loss due to light absorption by the solvent and the container, I is the laser power intensity and A_λ_ is the absorbance of the sample at the wavelength λ nm of the laser.

hs can be calculated based on the following equation:

(4) $$\mathrm{hs}=\frac{m\;C_{\mathrm{water}}}{\tau_{s}}$$

where m is the mass of the solution containing the sample, C_water_ is the specific heat capacity of water and τ_s_ is the time constant of the system.

### 4.11. Cell Culture

HepG2 cells were cultured in DMEM complete medium containing 10% fetal bovine serum (FBS) and 1% penicillin/streptomycin. The incubator environment was 37 °C, 5% CO_2_.

### 4.12. Cytotoxicity Test

The cytotoxicity assay was determined using the CCK-8 method. HepG2 cells in the logarithmic growth phase were collected and inoculated in 96-well plates at a density of 1 × 10^5^ cells/mL per well. Three parallel replicate wells were set up for each group. The cells were cultured in a complete medium at 37 °C, 5% CO_2_ overnight. Then, the medium was changed by discarding the original medium and replacing it with fresh medium containing different concentrations of materials (0, 50, 100, 200 μg/mL) and incubated for 24 h. The materials were grouped into PBS, PBS + L, DOX, MIL−101(Fe), MD, MDTM, MDTM@P−Ag and MDTM@P−Ag + L. The PBS + L group and the MDTM@P−Ag + L group were treated with NIR laser irradiation at 660 nm, 500 mW and 808 nm, 2 W/cm^2^. CCK-8 reagent was added to each well protected from light after removing the medium from the previous step, and the optical density (OD) value of each well was measured at 450 nm using an enzyme marker after 0.5–2 h of incubation.

Cytotoxicity calculations were based on the following equation:

(5) $$\mathrm{Cell}\;\mathrm{Viability}\;(\%)=\frac{{\mathrm{OD}}_{\mathrm{sample}}\;-{\;\mathrm{OD}}_{\mathrm{blank}}}{{\mathrm{OD}}_{\mathrm{control}}\;-\;{\mathrm{OD}}_{\mathrm{blank}}}\;\times \;100\%$$

### 4.13. Intracelluar GSH Generation

The detection of intracellular GSH content was achieved using the reduced GSH kit, which was purchased from Beyotime Biotechnology (Shanghai, China). HepG2 cells were inoculated in 6-well plates at a certain density and incubated overnight at 37 °C under 5% CO_2_ conditions. On the following day, the medium was changed, and the same concentration (100 μg/mL) of the materials was added, and the incubation was continued for 12 h. The materials were grouped into PBS, MIL−101(Fe), MDTM@P−Ag and MDTM@P−Ag + L. The MDTM@P−Ag + L group was irradiated with an NIR laser of 660 nm, 500 mW and 808 nm, 2 W/cm^2^. At the end of the incubation, the old medium was removed, and the cells were washed twice with PBS buffer. The cells were digested with trypsin and resuspended in 2 mL of PBS buffer, and the supernatant was collected by centrifugation for the detection of intracellular GSH content after lysis.

### 4.14. Intracelluar ROS Detection

To reveal the level of reactive oxygen species (ROS) generation in the cells after treatment with different materials, the fluorescent probe 2′,7′-dichlorofluorescein diacetate (DCFH-DA) was used for the detection of the generated ROS. HepG2 cells were inoculated in 6-well plates at a suitable density and cultured overnight. Then, a complete medium containing different materials (PBS, MIL−101(Fe), MD, MDTM, MDTM@P−Ag and MDTM@P−Ag + L) was incubated with different groups of cells for 4 h. The media containing the materials were removed and washed twice with PBS buffer. The cells in the MDTM@P−Ag + L group were treated with 660 nm laser irradiation before incubation, with the addition of the DCFH-DA probe. Incubation was performed for 15–30 min after the addition of the DCFH-DA probe. Subsequently, the intensity of green fluorescence in the cells was observed under a fluorescence microscope.

### 4.15. JC-1 Assays

The therapeutic effect of MDTM@P−Ag on HepG2 cells was further evaluated using JC-1 fluorescent dye. HepG2 cells in the logarithmic growth phase, with good growth conditions and suitable density, were inoculated in 6-well plates overnight. Then, the cells were cultured in a complete medium with the same concentration of different materials (PBS, MIL−101(Fe), MD, MDTM, MDTM@P−Ag and MDTM@P−Ag + L). After incubation, the old medium was discarded, and the cells of each group were washed with PBS buffer three times. Among them, the cells in the MDTM@P−Ag + L group were treated with NIR laser irradiation at 660 nm and 500 mW, and 808 nm at 2 W/cm^2^. After incubation with JC-1 for 15–30 min, the cells were washed with PBS buffer, and the fluorescence intensity in the cells was observed by fluorescence microscopy.

### 4.16. Statistical Analysis

All the data in the paper were analyzed by OriginPro 2021. The data are presented as the means ± standard deviation (SD). The statistical significance was analyzed by one-way analysis of variance (ANOVA). The *p*-value was considered significant at * *p* < 0.05, ** *p* < 0.01 and *** *p* < 0.001.

## Data Availability

The original contributions presented in the study are included in the article/Supplementary Material, further inquiries can be directed to the corresponding authors.

## Supplementary Materials

The following supporting information can be downloaded at: https://www.mdpi.com/article/10.3390/ph17060812/s1, Figure S1: SEM image of MDT. Figure S2: (**A**) MDTM@P−Ag NPs dispersed in deionized water, PBS, DMEM medium, and DMEM+10% FBS complete medium were incubated for 6 days, respectively. (**B**) The DLS size changes of MDTM@P−Ag NPs dispersed for 6 days in different media (n = 3). Figure S3: XRD patterns of MDTM, MDTM@P−Ag. Figure S4: XPS spectrum of MDTM@P−Ag. Figure S5: The N2 adsorption-desorption isotherms of MIL−101(Fe) and MD. Figure S6: The UV-vis absorption spectra of TMB with different materials at pH = 6.5: (**A**) MIL−101(Fe), (**B**) MDTM, (**C**) MDTM@P−Ag. (**D**) Time-dependent TMB UV-vis absorption spectra of the TMB+H2O2+MIL−101(Fe) mixture. (**E**) Time-dependent TMB UV-vis absorption spectra of the TMB+H2O2+MDTM mixture. (**F**) Time-dependent TMB UV-vis absorption spectra of the TMB+H2O2+MDTM−Ag mixture. Figure S7: The UV-vis absorption spectra of DTNB at different concentrations of MIL−101(Fe) treated with GSH.
