# Supplementary material for: Iron-Based Metal-Organic Frameworks as Multiple Cascade Synergistic Therapeutic Effect Nano-Drug Delivery Systems for Effective Tumor Elimination
Source: Pharmaceuticals (Basel). 2024 Jun 20;17(6):812. doi: 10.3390/ph17060812 (PMC11206809; doi:10.3390/ph17060812)
Supplement: Supplementary file 1 [file pharmaceuticals-17-00812-s001.zip › pharmaceuticals-3037706-supplementary.pdf]

Iron-based MOFs as Multiple Cascade Synergistic Therapeutic Effect Nano-Drug Delivery System for Effective Tumor Elimination

Materials

Ferric chloride hexahydrate ($FeCl_3 \cdot 6H_2O$, ≥98%), 2-aminoterephthalic acid ($BDC\text{-}NH_2$, 98%), N,N-Dimethylformamide DMF, 1-Ethyl-(3-dimethylaminopropyl)carbodiimide hydrochloride (EDC), N-hydroxysuccinimide (NHS), 5,10,15,20-tetrakis(4-carboxyphenyl)porphyrin (TCPP) were purchased from Shanghai Aladdin Biochemical Technology Co., LTD. Doxorubicin hydrochloride (DOX) and dopamine hydrochloride were purchased from Shanghai Macklin Biochemical Technology Co., LTD. DMEM, fetal bovine serum, trypsin, Penicillin/Streptomycin Dual Antibody were purchased from Nanjing Wisent Co., LTD.

Instruments

Scanning electron microscope (SEM, Sigma-300, Zeiss, UK) at 5 kV was used to observe the surface morphology of the samples. X-ray diffraction (XRD, Rigaku D/MAX 2500 V, Japan) with Cu Kα radiation (100 mA, 40 kV) at a scanning rate of 5 °/min^{-1} was used to characterize and analyze the crystal structure of the samples. The elemental composition and valence states of the samples were analyzed by X-ray photoelectron spectroscopy (XPS, Axis Ultra DLD, England). The adsorption and desorption isotherms of nitrogen were obtained by using an automatic specific surface area analyzer (TriStar II 3020, USA), and the Brunauer-Emmett-Teller (BET) was used to obtain the corresponding specific surface area.

Supplementary Figures

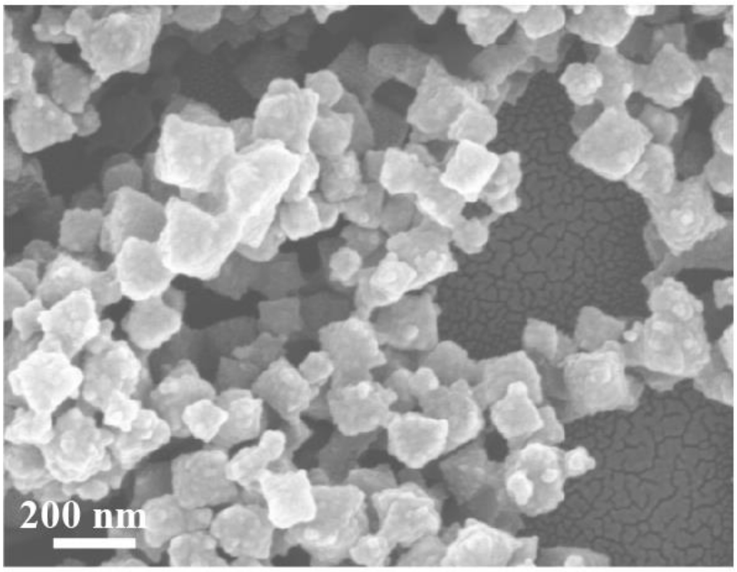

Figure S1. SEM image of MDT.

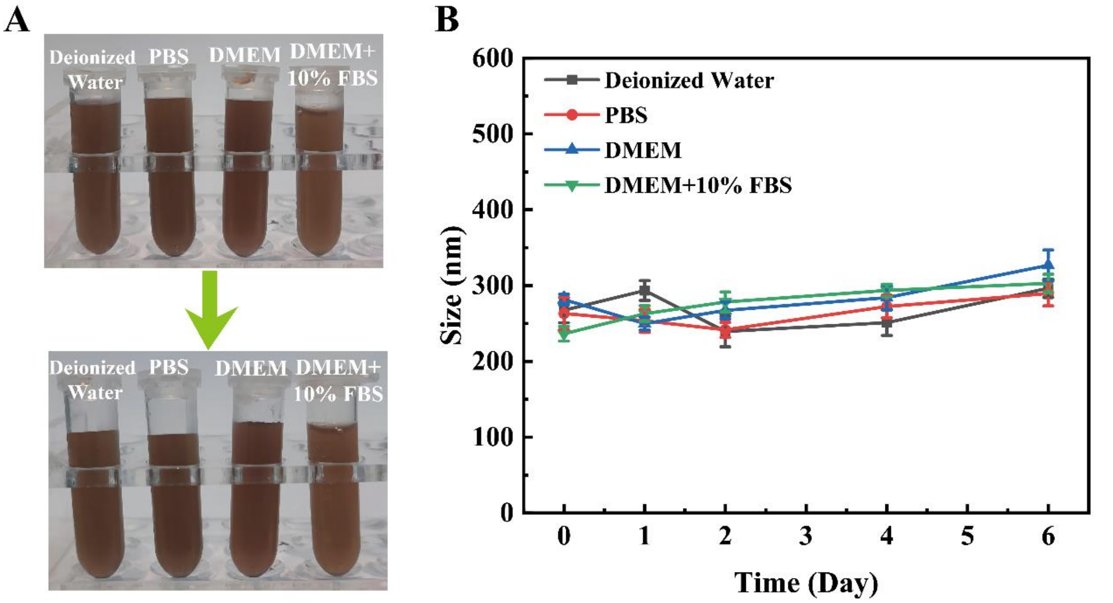

Figure S2. (**A**) MDTM@P-Ag NPs dispersed in deionized water, PBS, DMEM medium, and DMEM+10% FBS complete medium were incubated for 6 days, respectively. (**B**) The DLS size changes of MDTM@P-Ag NPs dispersed for 6 days in different media (n=3).

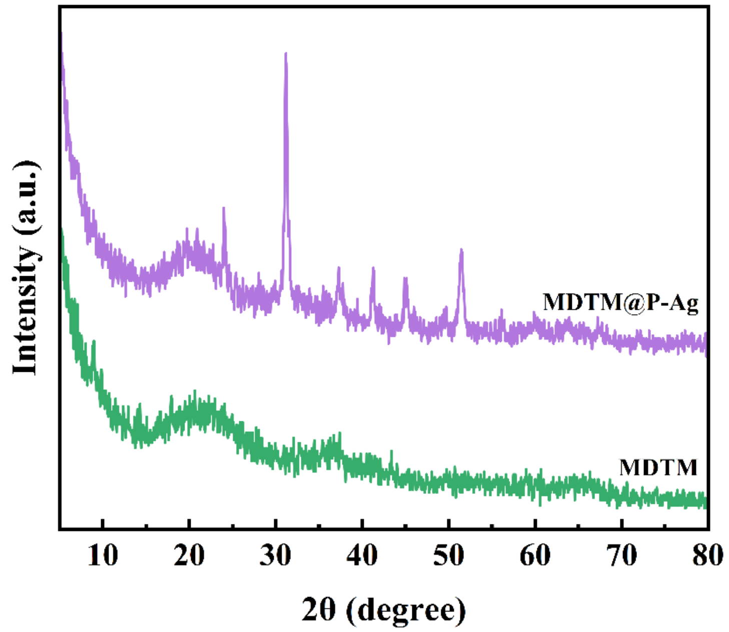

Figure S3. XRD patterns of MDTM, MDTM@P-Ag.

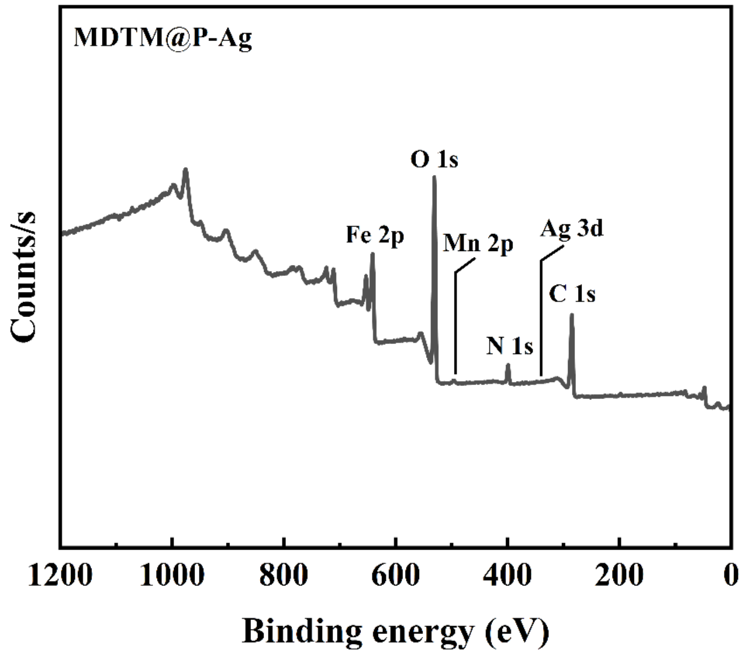

Figure S4. XPS spectrum of MDTM@P-Ag.

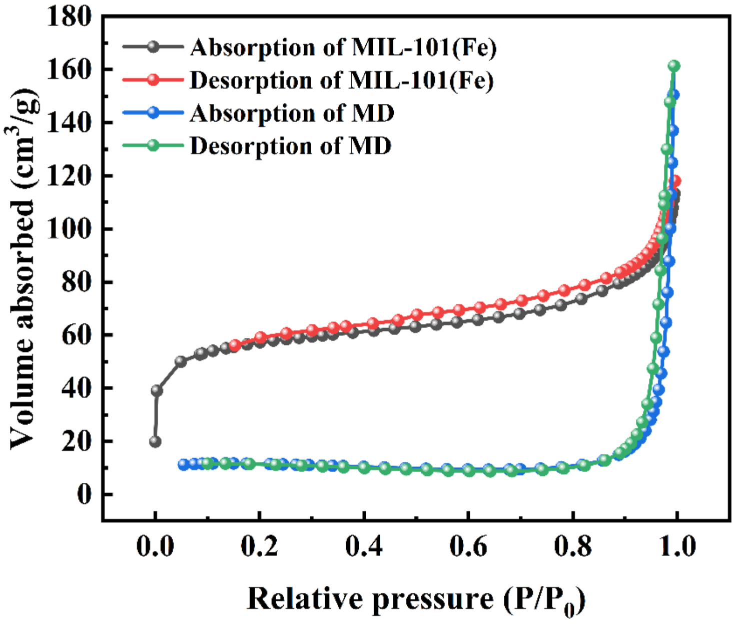

Figure S5. The N_2 adsorption-desorption isotherms of MIL-101(Fe) and MD.

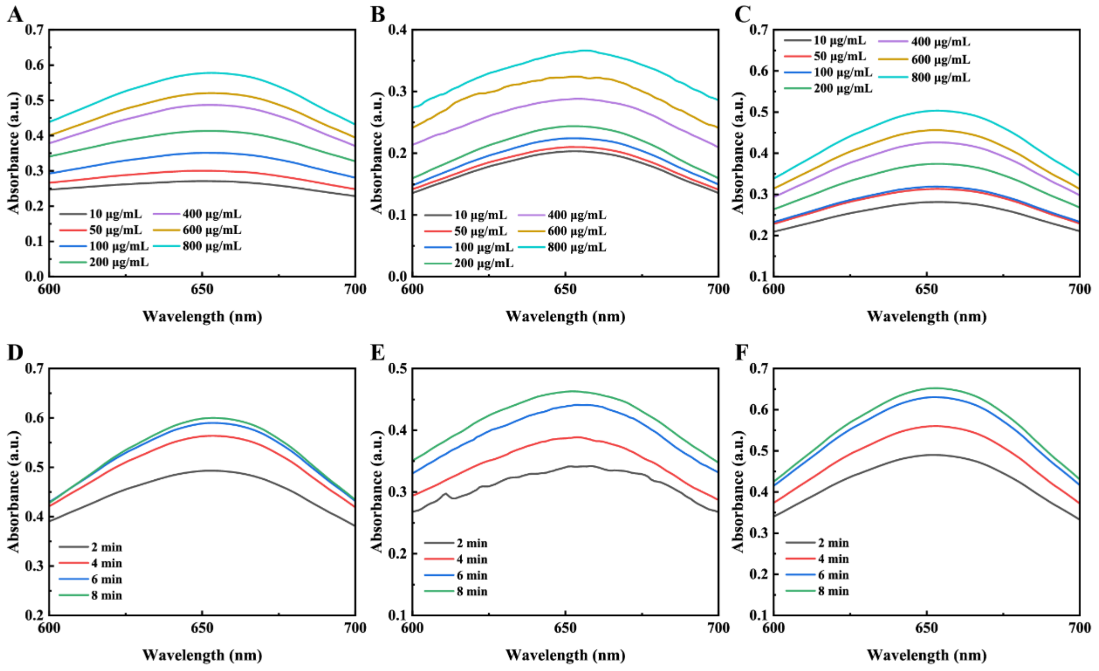

Figure S6. The UV-vis absorption spectra of TMB with different materials at pH=6.5: (**A**) MIL-101(Fe), (**B**) MDTM, (**C**) MDTM@P-Ag. (**D**) Time-dependent TMB UV-vis absorption spectra of the TMB+H_2O_2+MIL-101(Fe) mixture. (**E**) Time-dependent TMB UV-vis absorption spectra of the TMB+H_2O_2+MDTM mixture. (**F**) Time-dependent TMB UV-vis absorption spectra of the TMB+H_2O_2+MDTM-Ag mixture.

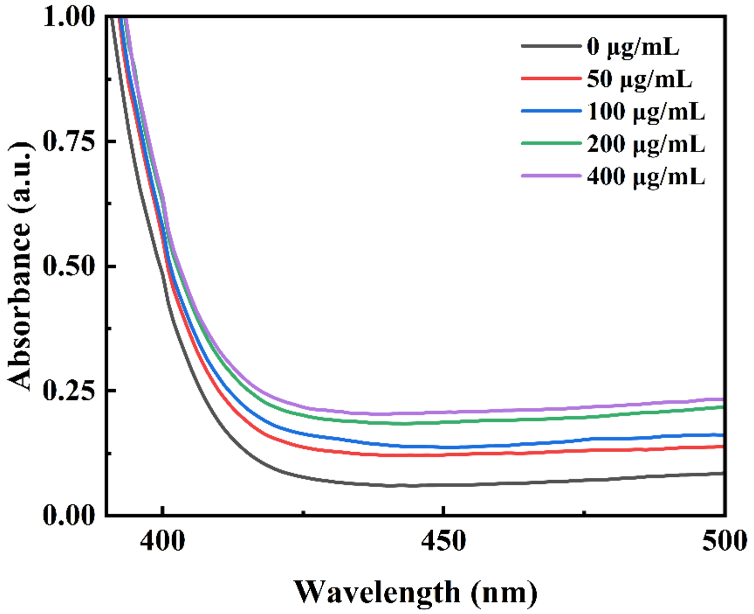

Figure S7. The UV-vis absorption spectra of DTNB at different concentrations of MIL-101(Fe) treated with GSH.
